# The effect of model selection on cost-effectiveness research: a comparison of kidney function-based microsimulation and disease grade-based microsimulation in chronic kidney disease modeling

**DOI:** 10.1186/s12911-018-0678-7

**Published:** 2018-11-09

**Authors:** Shusuke Hiragi, Hiroshi Tamura, Rei Goto, Tomohiro Kuroda

**Affiliations:** 1 Division of Medical Information Technology and Administration Planning, Kyoto University Hospital, 54 Kawaharacho, Shogoin, Sakyo-ku, Kyoto, 606-8507 Japan; 2 Department of Nephrology, Kyoto University Hospital, 54 Kawaharacho, Shogoin, Sakyo-ku, Kyoto, 606-8507 Japan; 3 Graduate School of Business Administration, Keio University, 2-33-28 Hiyoshi Honcho, Kohoku-ku, Yokohama, Kanagawa 223-8526 Japan; 4 Keio Business School, Keio University, 2-33-28 Hiyoshi Honcho, Kohoku-ku, Yokohama, Kanagawa 223-8526 Japan

**Keywords:** Chronic kidney disease, Health economics, Cost effectiveness analysis, Disease modeling

## Abstract

**Background:**

Cost effectiveness research is emerging in the chronic kidney disease (CKD) research field. Especially, an individual-level state transition model (microsimulation) is widely used for these researches. Some researchers set CKD grades as discrete health states, and the transition probabilities between these states were dependent on the CKD grades (disease grade-based microsimulation, MSM-dg), while others set estimated glomerular filtration rate value which determines the severity of CKD as a main variable describing patients’ continuous status (kidney function-based microsimulation, MSM-kf). MSM-kf seems to reflect the real world more precisely but is more difficult to implement. We compared the calculation results of these two microsimulation models to evaluate the effect of model selection on CKD cost-effectiveness analysis.

**Methods:**

We implemented simplified MSM-dg and MSM-kf emulating natural course of CKD in general, and compared models using parameters derived from an IgA nephropathy cohort. After checking these models’ overall behavior, life-years, utilities, and thresholds regarding intervention costs below which the intervention is thought as dominant (V0) or cost-effective (V1) were calculated. In addition, one-way and probabilistic sensitivity analyses were performed.

**Results:**

With base-case parameters, the calculated life-years was shorter in MSM-dg (73.89 vs. 75.80 years) while the thresholds were almost equal (86.87 vs. 90.43 (V0), 132.29 vs. 146.25 [V1 in 1000 USD]) compared to MSM-kf. Sensitivity analyses showed a tendency of the MSM-dg to show shorter results in life-years. V0 and V1 were distributed by approximately ±100,000 USD (V0) and ± 150,000 USD (V1) between models.

**Conclusions:**

Estimated cost-effectiveness thresholds by both models were not the same and its difference distributed too wide to be ignored. This result indicated that model selection in CKD cost-effectiveness research has large effect on their conclusions.

**Electronic supplementary material:**

The online version of this article (10.1186/s12911-018-0678-7) contains supplementary material, which is available to authorized users.

## Introduction

Chronic kidney disease (CKD), defined as impaired kidney function, is a very common disease that causes a high mortality rate and lowers quality of life (QOL) [1]. Progression of CKD to end-stage renal disease (ESRD) necessitates the renal replacement therapy (RRT, e.g. hemodialysis, peritoneal dialysis, or kidney transplantation), a costly [2] and lifelong treatment. As it is impossible to reverse renal impairment in CKD, in general, the effectiveness of management is judged by the extent to which disease progression is delayed.

CKD is said to progress gradually in a certain rate, depending on the underlying disease [3]. Various treatments developed to slow CKD progression have been investigated [4–6], and some researchers have evaluated the cost-effectiveness of these treatments [7–9]. One of the most utilized statistics for the measurement of cost-effectiveness is the incremental cost-effectiveness ratio (ICER), which is defined as the additional cost of a given treatment divided by the additional units of effects produced by the treatment. However, when we intend to analyze in a longer time horizon like CKD, which is lifelong disease, it is difficult to observe health utilities derived from treatments directly, because following patients for such a long time is typically not feasible.

Currently, computer simulations based on mathematical modeling are utilized to emulate patients’ prognoses in longer time horizon [10], and in particular, the decision analytic models are widely used. When evaluating a target intervention (e.g. drug, treatment), researchers first develop a disease prognosis model using parameters such as mortality rate, disease progression speed, cost, and QOL derived from epidemiological and in vitro data [11, 12]. Once the model is validated, the next step is to calculate the time courses of virtual patients using computer software and extract cost-effectiveness results.

In the decision analytic model, there are several ways to emulate the prognosis of a disease including decision tree analysis, and state transition modeling (STM). Decision tree analysis becomes unwieldy when many possible outcomes exist or when the follow-up period is very long [13] as with CKD. Comparably, STM is suitable for modeling lifelong diseases because these models are relatively simple and simulations can be carried out recursively. In STM, researchers define possible discrete health states that the patient can develop and input transition probabilities between them [14–16]. There are two types of STM: cohort-level STM and individual-level STM (microsimulation). Cohort-level STM simulates the average experience of the patients in a cohort, while individual-level STM, also called as microsimulation (MSM), simulates individual patient histories over time. Both STM models assume state transition occurs at once per predetermined time cycle [17].

CKD is defined by abnormalities of kidney structure or function, present for more than 3 months, with implications for health [18]. Structural abnormality is detected using urine sediment or imaging and so on, while functional abnormalities are defined by a glomerular filtration rate (eGFR) less than 60 mL/min/1.73 m^2^. The disease is classified into six grades according to eGFR (Table 1) [18], and sometimes the severity of kidney function impairment advances from grade 5 CKD to the condition named ESRD, in which patients are dependent on RRT for survival. eGFR is considered to reflect the total capacity of body waste filtration by tiny renal components known as nephrons. Two million nephrons are thought to exist in normal kidneys, which individually lose function for various reasons such as nephritis or diabetes. Therefore, eGFR is thought to decline steadily over time, which is supported by epidemiological study [19].

**Table 1:** Glomerular filtration rate (GFR) categories in chronic kidney disease (CKD). Patients with CKD G1 and G2 have normal eGFR value and have other evidence of kidney damages (e.g. proteinuria)

GFR category	GFR (mL/min/1.73 m^2)	Terms
G1	≥90	Normal or high, with evidence of kidney damage
G2	60–89	Mildly decreased, with evidence of kidney damage
G3a	45–59	Mildly to moderately decreased
G3b	30–44	Moderately to severely decreased
G4	15–29	Severely decreased
G5	< 15	Kidney failure

*CKD* Chronic kidney disease, *G* Grade, *GFR* Glomerular filtration rate, *eGFR* Estimated glomerular filtration rate

STM basically assumes discrete health statuses which patients can become. Therefore, researchers have used CKD grades as discrete health states between which transition probabilities are dependent on these grades (hereinafter referred to as disease grade-based microsimulation, MSM-dg). A research group also tried to find out these transition probabilities between these states in general population [20]. MSM-dg is easy to implement with commercially available software, but from the clinical viewpoint, the assumption that CKD stages are discrete states and transition probabilities are constant within a certain grade is unrealistic because severity of the disease is defined by eGFR, which is continuous variable declining constantly. In contrast, STM using eGFR as a constantly changing variable (hereinafter referred to as kidney function-based microsimulation, MSM-kf) has also been implemented [21], in spite of its difficulty in implementation, and it seems to emulate real world more precisely. Aforementioned models are based on different assumptions; health states are discrete or continuous, and thus it is possible that they show different results for the same research topic. So far, there is no research examining the difference in calculation results between these models, which can affect the conclusion of cost-effectiveness research. Hence, we aimed to compare the calculation results of these modeling methods and evaluate the effect of model selection on research conclusion to provide information for future CKD health economics investigators.

## Methods

We implemented simplified MSM-dg and MSM-kf models that were not intended to describe a specific kidney disease but instead to simulate the natural course of CKD in general, for use in comparing results derived from them. The details of development and the comparisons are described below.

### Implementing disease grade-based microsimulation (MSM-dg)

**Fig. 1:**
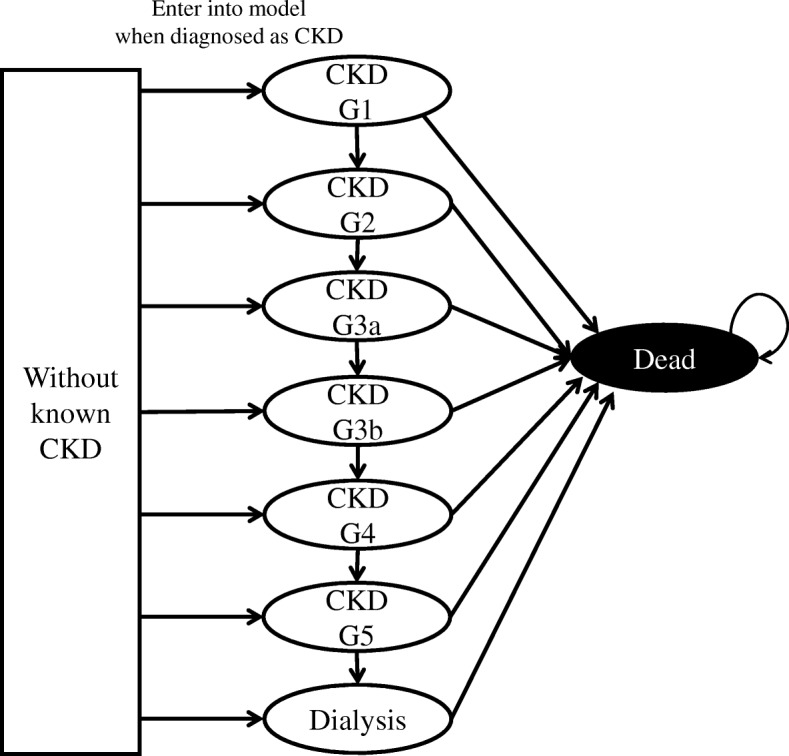
State transition diagram of disease grade-based microsimulation (MSM-dg). The transition probabilities between states depend on CKD grades

We implemented individual-level STM (microsimulation, MSM) in which we set CKD grades as health states and assumed transition probabilities between states were dependent on these grades only (Fig. 1), adhering to conventional STM assumption. Once the kidney is damaged, the damage generally cannot be fully cured. Thus, the target population in cost-effectiveness research for treating kidney disease can be assumed to have grade 1 or more advanced CKD stages, or ESRD. With this model, the virtual cohort’s time course was computed as shown in the flowchart on Additional file 1: Figure S1. Mortality rates were set as a function of age, sex, and CKD grade and that of patients in the predialysis and dialysis periods were set separately. Time cycle was set as 1/4 years based on the Japanese CKD guideline which recommends every CKD patient should be followed-up with eGFR measurement every 3 months [22]. In general, visiting an internal medicine clinic once every 3 months is common practice [23]. We repeated the process for each individual in the virtual cohort and acquired historical data including each individual’s health state, accumulated cost, and utility.

### Implementing kidney function-based microsimulation (MSM-kf)

**Fig. 2:**
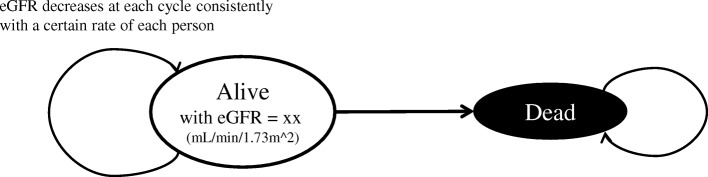
State transition diagram of kidney function-based microsimulation (MSM-kf). The transition probabilities between states depend on eGFR value which constantly decreases at each time cycle with a certain rate

Next, we implemented MSM-kf in which we set only two health states (alive and dead), but the alive state has eGFR value as constantly declining variable (Fig. 2). The decline rates were sometimes said to be non-linear [24], but we assumed widely-believed constant decline rate for simplicity. The transition probability between alive and dead states is dependent on CKD grades, which is determined only by the eGFR value. We assumed the decline rates are different by person to person. The mean and standard deviations of the rate are already well examined [25–27], and we utilized these data when developing our model. We assumed a log-normal distribution for the rates of eGFR decline [28], with assumption that no patient had continuously increasing eGFR value.

The simulation flowchart is shown in Additional file 2: Figure S2. Mortality rates, accumulated costs, and utilities were analyzed as described for the MSM-dg implementation. The conversion from eGFR to CKD grade was based on the aforementioned KDIGO definition, and we assumed that patients with an eGFR < 7 mL/min/1.73 m^2 were ESRD who required RRT according to the IDEAL study [29]. We also set time cycle as 1/4 years with the same reason explained in MSM-dg implementation section. The historical data of each patient was obtained using methods similar to those described for MSM-dg, and we calculated several parameters discussed below.

### Parameter translation for model comparison

**Fig. 3:**
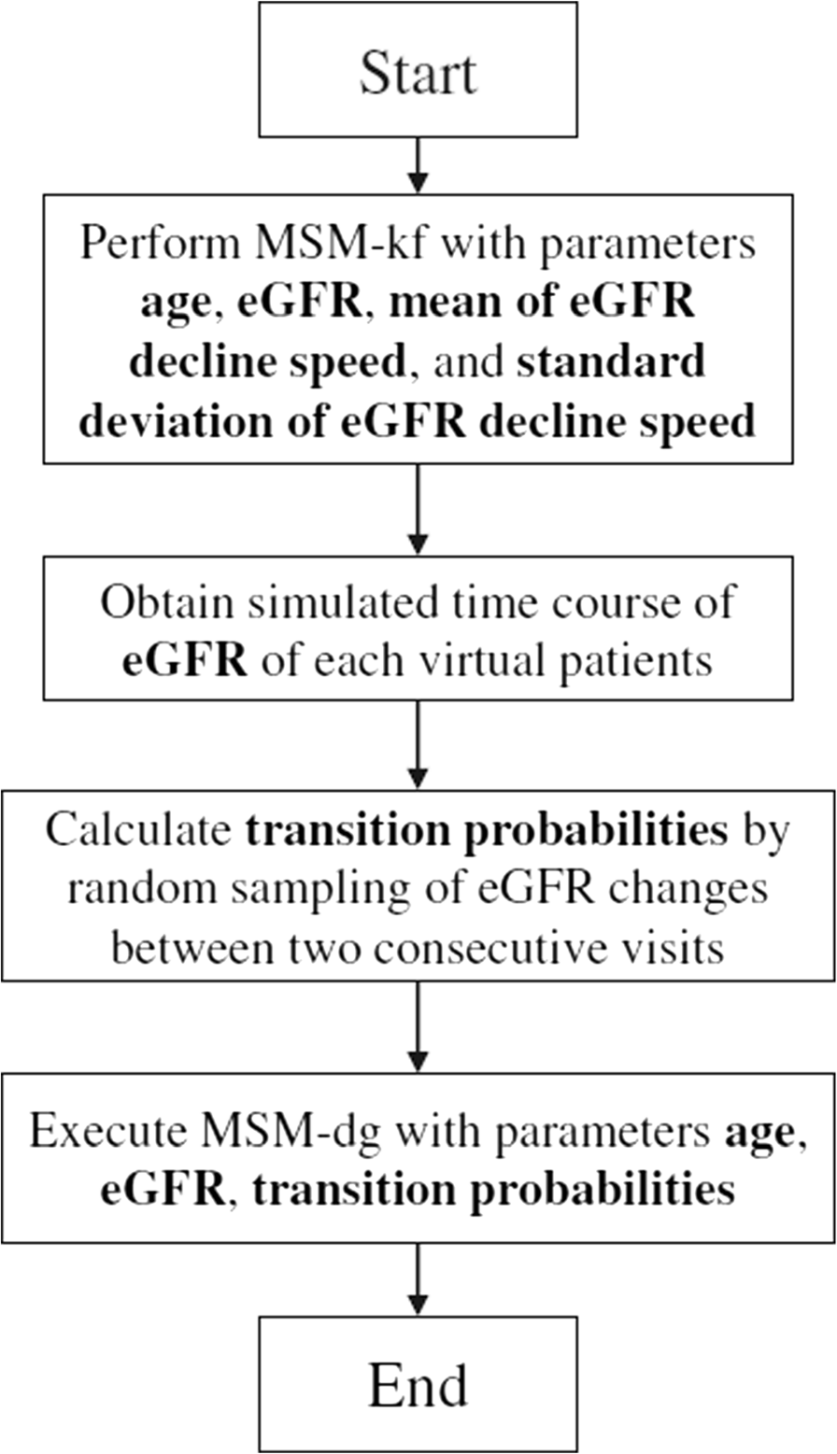
Parameter adjustment and comparison of MSM-dg and MSM-kf flowchart

When comparing MSM-dg and MSM-kf, it is essential to translate parameters. In particular, transition probabilities in MSM-dg and eGFR decline rates in MSM-kf. Generally, previous researchers obtained the transition probabilities for MSM-dg from medical records [20, 30]. That is, they compared the eGFR of patients at two visits and calculated the fraction of those who proceeded to the next grade among all patients with a particular CKD grade. On the contrary, investigators applying MSM-kf used eGFR decline rates obtained from previous cohort studies [21, 31]. Therefore, we at first performed our MSM-kf with parameters obtained from existing epidemiological data. Next, from the historical eGFR data acquired with the MSM-kf, we randomly sampled the grade transition between two consecutive visits and estimated the transition probabilities to emulate the method described above to acquire transition probabilities. Finally, we inputted the calculated transition probabilities for MSM-dg (Fig. 3). By implementing the aforementioned translation, we could calculate transition probabilities from eGFR decline slope, resulting in comparison between MSM-dg and MSM-kf with same conditions.

### Comparison of estimation results with MSM-dg and MSM-kf

We compared MSM-dg and MSM-kf to evaluate the differences in the calculation results. In this study, we set MSM-kf as reference model, considering its potential to reflect the reality more than MSM-dg. As base case analysis parameters, we used data from prior epidemiological study on IgA nephropathy, a major cause of CKD [32]. Clinically, utility changes from the disease and natural course represent those of typical CKD and cost structure may be the same, suggesting the data was suitable for our purpose. The study, named VALIGA [33], consisted of patients from 13 European countries. Adhering the data, we made our virtual cohort include equal numbers of 37-year old men and women with an eGFR of 76 mL/min/1.73 m^2^. Mortality rates were acquired from general population of Ontario [34–36], Canada and dialysis patients from Japan [37] due to limited available data (Detail is described in supplementary material provided as Additional file 3). The study showed immunosuppressant therapy for IgAN can slow the rate of eGFR decline from − 2.2 ± 6.5 to − 1.3 ± 8.5 mL/min/1.73 m^2^/year. We set the initial parameters as shown in Table 2. At first, we checked these models’ behavior by means of Kaplan-Meier plot of renal survival rates. Renal survival rate is a ratio of patients not requiring RRT at a point of certain time, which is common indicator in nephrology research. Afterwards, we calculated and compared unadjusted life years, discounted quality-adjusted life-years (QALYs), costs, and new parameters defined below with both MSM-dg and MSM-kf.

**Table 2:** Parameters of base case and sensitivity analyses. There were no CKD 1 patients as the initial eGFR was below 90 mL/min/1.73 m^2. Hence, we did not perform sensitivity analyses of mortality rate, utility, and cost for CKD 1 patients

Parameters	Baseline values	Sensitivity analyses		Ref.
		Lower limit	Upper limit	
Mean of eGFR decline speed (mL/min/1.73 m^2/year)	2.2	0.1	10	[33]
Standard Deviation of eGFR delcline speed (mL/min/1.73 m^2/year)	6.5	0.1	10	[33]
Initial age (years)	37	30	60	[33]
Initial eGFR (mL/min/1.73 m^2)	73	30	75	[33]
Baseline mortality rate of predialysis patients	3.338 × 10^−5^ × *e*^0.091 × *age*^(male) 1.615 × 10^−5^ × *e*^0.098 × *age*^(female)	× 0.8	× 1.2	[34, 35]
Mortality rate of CKD 1 patients	Baseline × 1	× 0.9	× 1.1	[36]
Mortality rate of CKD 2 patients	Baseline × 1	× 0.9	× 1.1	[36]
Mortality rate of CKD 3a patients	Baseline × 1.2	× 1.1	× 1.3	[36]
Mortality rate of CKD 3b patients	Baseline × 1.8	× 1.7	× 1.9	[36]
Mortality rate of CKD 4 patients	Baseline × 3.2	× 3.0	× 3.4	[36]
Mortality rate of CKD 5 patients	Baseline × 5.9	× 5.6	× 6.2	[36]
Mortality rate of dialysis patients	1.32 × 10^−3^ × *e*^0.060 × *age*^	× 0.8	× 1.2	[36]
Utility of CKD 1 patients	1	0.9	1	[12]
Utility of CKD 2 patients	0.9	0.8	1	[12]
Utility of CKD 3a patients	0.87	0.77	0.97	[12]
Utility of CKD 3b patients	0.85	0.75	0.95	[12]
Utility of CKD 4 patients	0.85	0.75	0.95	[12]
Utility of CKD 5 patients	0.85	0.75	0.95	[12]
Utility of dialysis patients	0.72	0.62	0.82	[12]
Cost of CKD 1 (1000 USD / year)	1.6	0.8	3.2	[46]
Cost of CKD 2 (1000 USD / year)	1.7	0.9	3.4	[46]
Cost of CKD 3a (1000 USD / year)	3.5	1.8	7.0	[46]
Cost of CKD 3b (1000 USD / year)	3.5	1.8	7.0	[46]
Cost of CKD 4 (1000 USD / year)	12.7	6.4	25.4	[46]
Cost of CKD 5 (1000 USD / year)	12.7	6.4	25.4	[46]
Cost of dialysis patients (1000 USD / year)	84.6	42.3	169.1	[2]
Improve rate of eGFR decline slope	0.59	0.1	0.9	[33]
Annual discount rate for costs	0.03	0	0.1	
Annual discount rate for utilities	0.03	0	0.1	

*CKD* Chronic kidney disease, *eGFR* Estimated glomerular filtration rate, *Ref* Reference

**Fig. 4:**
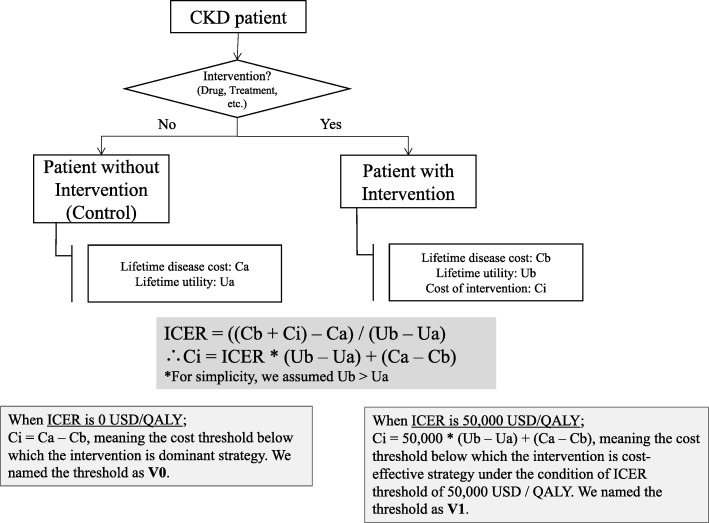
Pictorial representation of definition of V0 and V1 values

Here, we introduced two new parameters indicating cost thresholds below which an interventions of interest are dominant strategy, and cost-effective (Fig. 4). In particular: the maximum present value of the cost of an intervention below which the intervention can be dominant over the control (named V0 in this study) under the assumption that utility increases by the intervention, and the maximum present value of the cost of an intervention below which the intervention can be thought as cost-effective (V1). For this study, we assumed the threshold of cost-effectiveness was 50,000 USD / QALY adhering to prior literature [38]. The formulas used to calculate V0 and V1 are:

$$ \mathrm{V}0=\mathrm{Ca}-\mathrm{Cb}\ \left(\mathrm{under}\ \mathrm{the}\ \mathrm{assumptions}\ \mathrm{of}\ \mathrm{Ub}>\mathrm{Ua}\right)\ \mathrm{and}\ \mathrm{V}1=50,000\times \left(\mathrm{Ub}-\mathrm{Ua}\right)+\left(\mathrm{Ca}-\mathrm{Cb}\right) $$

Where Ca = total cost of disease without intervention, Cb = total cost of disease with intervention (excluding intervention costs), Ua = total utility without intervention, and Ub = total utility with intervention. When Ca > Cb + cost of intervention, the intervention is dominant over the control. Hence, V0 = Ca – Cb. When (Cb + cost of intervention – Ca) / (Ub – Ua) < 50,000, the intervention will be seen as cost-effective. Hence, V1 = 50,000 × (Ub – Ua) + (Ca – Cb).

These variables represent the value we can pay for the intervention (detail is shown in Fig. 4). By calculating them with MSM-dg and MSM-kf, we can evaluate the effect of modelling methods on researchers’ conclusion whether a new expensive intervention is cost effective or not.

### Sensitivity analysis

There was unavoidable uncertainty in the parameters extracted from the VALIGA cohort, which provided only cohort-level information. Therefore, we performed one-way sensitivity analysis to overcome this limitation. We altered eGFR (30–90 mL/min/1.73 m^2), initial age (30–60 years old), mean eGFR decline rate (0.5–10.0 mL/min/1.73 m^2/year), standard deviation of the eGFR decline rate (0.5–10.0 mL/min/1.73 m^2/year), and other variables listed in Table 2 and calculated difference between estimated life years, utilities, costs, V0 and V1.

Researchers may be interested in not only IgAN, but also other diseases causing CKD, in other cohorts. Thus, we also performed probabilistic sensitivity analysis of these parameters and plotted the estimated life years, utilities, costs, and V0 and V1, from both models.

We used the Numpy and Math libraries. (Source codes are provided as Additional files 4 and 5). For visualization, we used R 3.4.2 software [39] and “survminer” package [40] to draw the Kaplan-Meier curve.

## Results

### Comparison of models

**Fig. 5:**
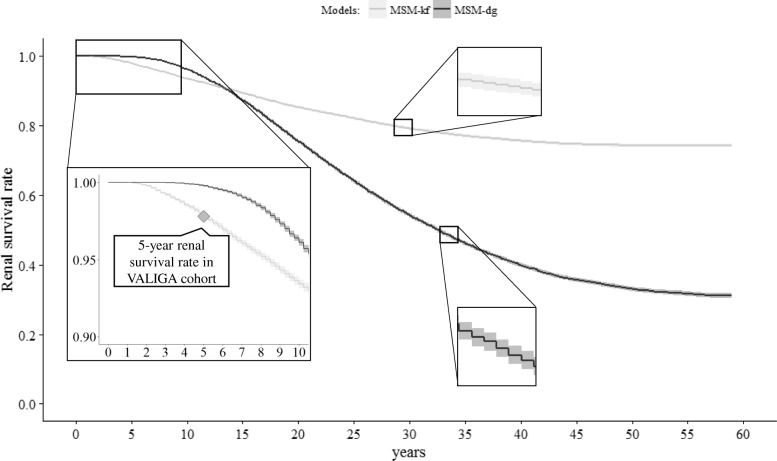
Kaplan-Meier curve of renal survival rates calculated with MSM-dg and MSM-kf. The black line represents renal survival rate calculated with MSM-kf; gray line with MSM-dg. Light color bands represents 95% confidence intervals

Figure 5 shows the Kaplan-Meier plot of the renal survival rates in both models. That of 5-years follow up in VALIGA cohort was also shown in zoomed area in lower left for comparison. Renal survival rates at 5 years were 99.8% in MSM-dg, 97.8% in MSM-kf, and 97.8% in VALIGA cohort.

The estimated life-years determined using MSM-dg were 73.89 ± 12.14 years (control) and 75.80 ± 12.82 years (immunosuppressant therapy). The estimated life-years using MSM-kf were 76.35 ± 12.44 years (control) and 78.80 ± 12.62 years (immunosuppressant therapy). The expected utilities (discounted) from the starting point calculated by MSM-dg were 19.43 ± 4.06 QALYs (control) and 20.00 ± 4.33 QALYs (immunosuppressant therapy). The expected utilities calculated by MSM-kf were 20.34 ± 4.08 QALYs (control) and 21.12 ± 4.08 QALYs (immunosuppressant therapy). The present values of expected lifetime costs calculated by MSM-dg were 286.85 ± 350.27 thousand dollars (control) and 213.42 ± 365.60 thousand dollars (immunosuppressant therapy, excluding intervention costs). The present values of expected lifetime costs calculated by MSM-kf were 199.98 ± 298.13 thousand dollars (control) and 122.99 ± 265.59 thousand dollars (immunosuppressant therapy, excluding intervention costs). The V0 values calculated from acquired data were 86,870 USD (MSM-dg) and 90,430 USD (MSM-kf). The V1 values calculated from the same data were 132,290 USD (MSM-dg) and 146,250 USD (MSM-kf) (Table 3). In base cases, the MSM-dg results showed shorter life-years, lower utilities, and greater costs than MSM-kf did. As a result, MSM-dg showed smaller result in both V0 and V1 compared to MSM-kf in this circumstance.

**Table 3:** Calculation results of base cases. Standard deviations are shown in parentheses

	MSM-dg		MSM-kf	
	Immunosuppressant therapy	Control	Immunosuppressant therapy	Control
Life years (years)	76.35 (± 12.44)	73.89 (± 12.14)	78.80 (± 12.62)	75.80 (± 12.82)
Utility (years)	20.34 (± 4.08)	19.43 (± 4.06)	21.12 (± 4.08)	20.00 (± 4.33)
Cost (1000 USD)	199.98 (± 298.13)	286.85 (± 350.27)	122.99 (± 265.59)	213.42 (± 365.60)
V0 (1000 USD)	86.87		90.43	
V1 (1000 USD)	132.29		146.25	

*MSM-dg* Disease grade-based microsimulation, *MSM-kf* Kidney function-based microsimulation, *V0* boundary below which the intervention is dominant; *V1* boundary below which the intervention is cost-effective. V0 and V1 are defined in the main text

### Sensitivity analysis

The results of the one-way sensitivity analysis revealed that MSM-dg showed shorter and lower life-years and utilities than MSM-kf in most cases, respectively. Also similar to the base case analyses, the calculated costs determined using MSM-dg were greater by approximately 50,000–100,000 USD in most cases. The difference in V0 and V1 values did not show specific tendencies, and the differences distributed within approximately − 50,000 to 100,000 USD (V0) and − 100,000 to 150,000 USD (V1). The detailed result of one-way sensitivity analyses are shown in Additional file 6: Figure S3.

The probabilistic sensitivity analysis revealed that life-years and utilities calculated with MSM-dg were shorter and lower, respectively, than those with MSM-kf in approximately two thirds of parameter sets. Costs calculated with MSM-dg were greater than costs calculated with MSM-kf in two thirds of cases. Regarding V0 and V1 values, the difference between both results distributed across zero almost equally. The vast majority of results (95%) were between − 91,300 USD and 75,700 USD (V0), and between − 108,300 USD and 98,600 USD (V1). The scatter plots of results of probabilistic sensitivity analyses are shown in Additional file 7: Figure S4.

## Discussions

We compared two microsimulation models of CKD that are utilized within the field of health economics research: we called them MSM-dg and MSM-kf. First, we implemented simplified models. The Kaplan-Meyer curves of calculated results declined steadily, indicating that our implementations did not deviate from the reality so much.

We introduced two new parameters, V0 and V1, for our base-case settings comparison because directly analyzing cost-effectiveness of currently available therapy has scarce meaning. That is, currently available, partially-effective immunosuppressant therapy for IgAN [41] costs only 1200 USD [42, 43], which is too inexpensive to analyze cost-effectiveness with commonly-applied 50,000 USD/QALY threshold. We defined V0 as the difference between the total estimated costs of the control and intervention groups excluding the intervention cost. When the disease costs of the control group exceed those of the intervention group, V0 is positive and identical to the maximum present value of the cost of the intervention below which the intervention is dominant strategy over the control. Likewise, V1 represents the maximum present value of the intervention cost under the condition that the cost-effectiveness threshold is 50,000 USD/QALY. The threshold determining cost-effectiveness is a topic of health economics research [44] but is outside the scope of this study. Current effort on drug discovery focuses on innovative and expensive ones which should be examined cost-effectiveness. It is quite possible that such an expensive drug for stopping CKD would be invented in near future. From the viewpoint of payers or societies, it is very important to estimate the threshold below which an intervention is cost-effective for future CKD interventions in the era of increasing medical costs. In this context, V0 and V1 express this threshold.

In base-case analysis, MSM-dg results showed shorter life years and QALYs, and larger costs compared to MSM-kf. V0 were 86,870 USD by MSM-dg and 90,430 USD by MSM-kf, meaning that investigators who use MSM-dg will conclude an intervention is dominant strategy when the present value of the intervention cost is below 86,870 USD while those who use MSM-kf do so if the present value is below 90,430 USD. If the actual cost is between 86,870 and 90,430USD, conclusion becomes different due to selection of modeling method. Likewise, if the actual cost is between 132,290 USD and 146,250 USD, investigators conclude differently about cost-effectiveness of the intervention in the condition of ICER threshold is 50,000USD / QALY. This result indicates that the conclusion whether an intervention is dominant strategy or cost-effective is easily changed by the selection of their modeling method. In base-case setting, investigators who use MSM-kf will conclude the intervention is cost-effective more likely than those who use MSM-dg.

Our one-way sensitivity analysis showed that MSM-dg showed shorter results in life-years (by approximately 1.5–3.0 years) and utilities compared to MSM-kf but did larger results in costs in most cases. V0 and V1 values did not show specific tendencies, but the results calculated by MSM-dg were different from those by MSM-kf by approximately − 50,000 to 100,000 USD (V0) and − 100,000 to 150,000 USD (V1). These results imply that even if the initial parameters were uncertain, the tendencies were applicable with regard to calculated life years, utilities, and costs. Difference in V0 and V1 distributed largely, indicating that the threshold for an intervention to be dominant or cost-effective can fluctuate within these margins based on the selection of modeling method. These fluctuation margins are three to five times greater than the GDP per capita in the United States [45]; an amount that is difficult to ignore.

We performed probabilistic sensitivity analysis for proving generalizability of our findings. The analysis showed similar results to one-way sensitivity analysis. The differences between the V0 values calculated using MSM-dg versus MSM-kf were in the range of − 91,300 USD and 75,700 USD in 95% of the results while that of V1 values ranged from − 108,300 USD and 98,600 USD. These amounts are also difficult to ignore.

The reason for this difference can be partially explained by the characteristics of MSM-dg which ignored time-dependent variety of patients within a single health states. A cohort with eGFR of 55 mL/min/1.73m^2^ (CKD G3a) at the beginning of a simulation, for instance, should have time-dependent transition probabilities to G3b because the fraction of patients with eGFR close to 45 mL/min/1.73 m2 (threshold between CKD G3a and G3b) will increase as the time goes, under the assumption of constant eGFR decline.

Policymakers can use cost-effectiveness studies to determine whether a given intervention is worth paying for. CKD is a common and lifelong disease with treatments available that may slow its progression to ESRD. Based on the results of our study, MSM-dg and MSM-kf based on different health states assumptions may produce different conclusions. In particular, compared to MSM-kf, MSM-dg showed shorter or smaller results in life-years and utilities, and estimated costs. The differences the between the V0 and V1 values were distributed bilaterally across zero, and the margins were relatively large. At a glance, MSM-kf emulates the real world more precisely from the clinical viewpoint. However, MSM-dg is easier to understand than MSM-kf, and it is more easily implemented using commercially available software. Simulation methods cannot avoid inaccuracy, and investigators must consider the biases inherent to the calculation methods they utilize. Our results may help clarify the biases derived from the selection of MSM-dg or MSM-kf for future CKD cost-effectiveness researchers.

This research has several limitations. First, we ignored major parameters affecting CKD progression such as race, ethnicity, and major cardiovascular risks for simplicity. Second, we did not consider kidney transplantation, which has a different cost structure than dialysis as RRT. Kidney transplantation is relatively inexpensive, and has become a considerable option for ESRD patients. This time we did not include kidney transplantation in our models to maintain simplicity, but it may be possible to include post-transplantation status into the models after reliable cost and utility data are accumulated. Third, we assumed constant eGFR decline rates, which is widely believed but sometimes pointed out to be incorrect. However, even when non-linear decline was assumed, our method of comparison could be applied even though it may become complicated, and similar difference would be possibly shown. Lastly, we only showed difference in calculation results, but we could not definitively determine which model is superior because of limited reliable accuracy indicators. Nevertheless, we believe this information is important.

## Conclusions

MSM-dg, based on a conventional discrete state transition assumption, tends to show smaller results in utilities and larger ones in costs compared to more-realistic MSM-kf, based on the assumption of continuous state change in CKD disease modeling. The selection of a disease modelling method in cost-effectiveness researches of CKD intervention causes difficult-to-ignore fluctuations in their conclusions.

## Additional files

Additional file 1: **Figure S1.** Implemented MSM-dg flowchart. Uniform random numbers were used for determination of live or death, and progression of CKD grade. Each period’s costs and utilities were added after a state transition was determined. (PPT 192 kb)

Additional file 2: **Figure S2.** Implemented MSM-kf flowchart. Log-normal random numbers with reported mean and standard deviation values were used as the patient’s constant eGFR decline rate. Similar to MSM-dg, a uniform random number was generated for determination of live or death. If the patient survives, his/her eGFR declined according to the previously assigned constant rate. Each period’s costs and utilities were added after a state transition was determined. (PPT 174 kb)

Additional file 3: **Figure S2.** Supplementary documents describing parameter determination in detail. (DOC 128 kb)

Additional file 4: Python source code for models implementation. Python source code which implements MSM-kf and MDM-dg models. (TXT 9 kb)

Additional file 5: Python source code for models comparison. Python source code which implements models comparison. Users have to modify the code to import python code provided as Additional file 4 (Python source code for models implementation). (TXT 3 kb)

Additional file 6: **Figure S3.** a-e Results of one-way sensitivity analyses. (PPT 132 kb)

Additional file 7: **Figure S4.** a-e Results of probabilistic sensitivity analyses. (PPT 3755 kb)

## Abbreviations

CKD: Chronic kidney disease
eGFR: Estimated glomerular filtration rate
ESRD: End-stage renal disease
ICER: Incremental cost-effectiveness ratio
IgAN: IgA nephropathy
MSM: Microsimulation
MSM-dg: Disease grade-based microsimulation
QALY: Quality-adjusted life-year
QOL: Quality of life
RRT: Renal replacement therapy
STM: State transition modelling
