# Supplementary material for: The effect of model selection on cost-effectiveness research: a comparison of kidney function-based microsimulation and disease grade-based microsimulation in chronic kidney disease modeling
Source: BMC Med Inform Decis Mak. 2018 Nov 9;18:94. doi: 10.1186/s12911-018-0678-7 (PMC6230230; doi:10.1186/s12911-018-0678-7)
Supplement: Supplementary file 1 — Figure S1. Implemented MSM-dg flowchart. Uniform random numbers were used for determination of live or death, and progression of CKD grade. Each period’s costs and utilities were added after a state transition was determined. (PPT 192 kb) [file 12911_2018_678_MOESM1_ESM.ppt]

## Slide 1
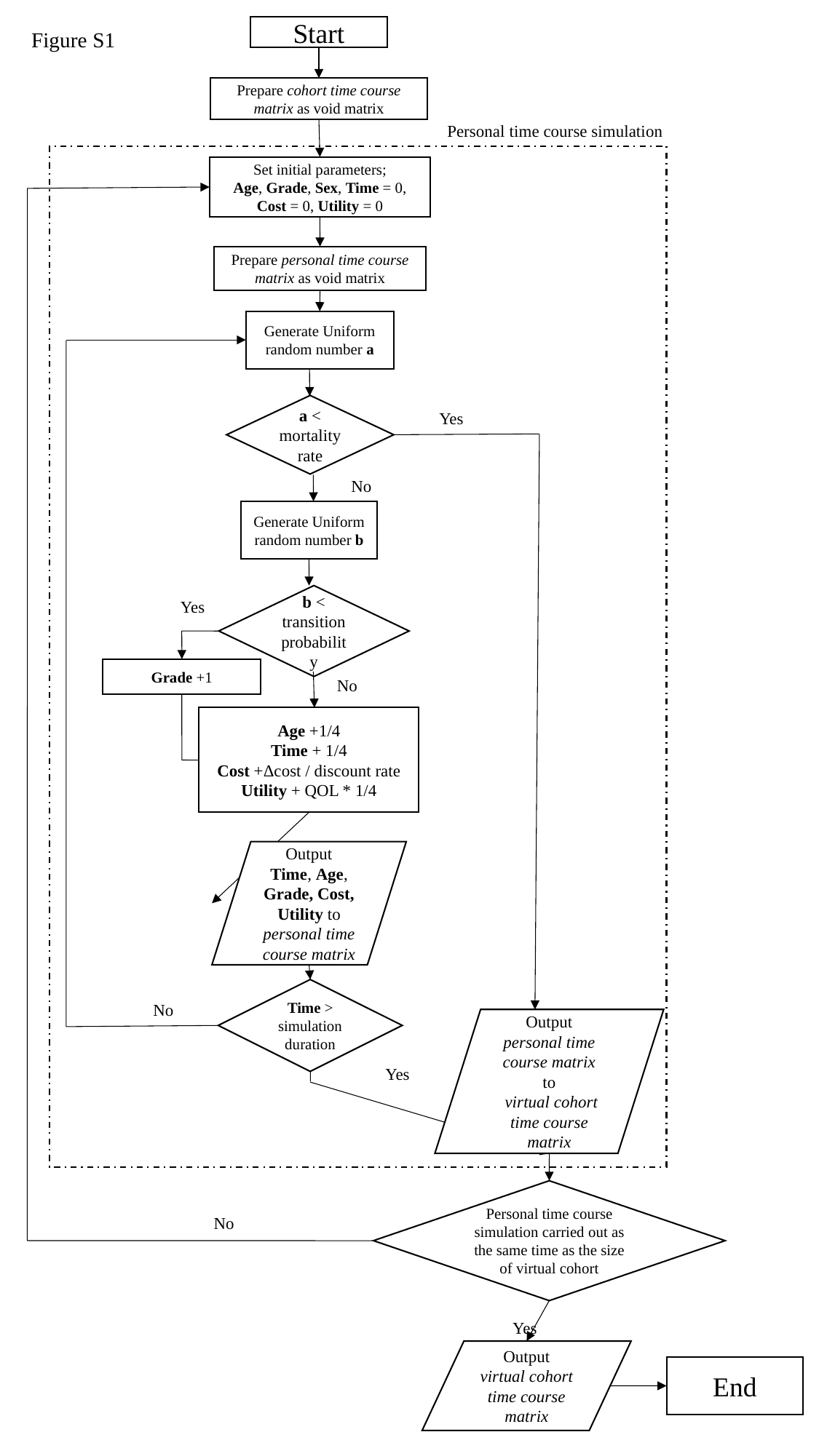

Start
Figure S1
Prepare cohort time course matrix as void matrix
Personal time course simulation
Set initial parameters;
Age, Grade, Sex, Time = 0, Cost = 0, Utility = 0
Prepare personal time course matrix as void matrix
Generate Uniform random number a
a < mortality rate
Yes
No
Generate Uniform random number b
b < transition probability
Yes
Grade +1
No
Age +1/4
Time + 1/4
Cost +Δcost / discount rate
Utility + QOL * 1/4
Output
Time, Age, Grade, Cost, Utility to personal time course matrix
Time > simulation duration
No
Output
personal time course matrix
to
 virtual cohort time course matrix
Yes
Personal time course simulation carried out as the same time as the size of virtual cohort
No
Yes
Output
virtual cohort time course matrix
End
