# Supplementary material for: The effect of model selection on cost-effectiveness research: a comparison of kidney function-based microsimulation and disease grade-based microsimulation in chronic kidney disease modeling
Source: BMC Med Inform Decis Mak. 2018 Nov 9;18:94. doi: 10.1186/s12911-018-0678-7 (PMC6230230; doi:10.1186/s12911-018-0678-7)
Supplement: Supplementary file 2 — Figure S2. Implemented MSM-kf flowchart. Log-normal random numbers with reported mean and standard deviation values were used as the patient’s constant eGFR decline rate. Similar to MSM-dg, a uniform random number was generated for determination of live or death. If the patient survives, his/her eGFR declined according to the previously assigned constant rate. Each period’s costs and utilities were added after a state transition was determined. (PPT 174 kb) [file 12911_2018_678_MOESM2_ESM.ppt]

## Slide 1
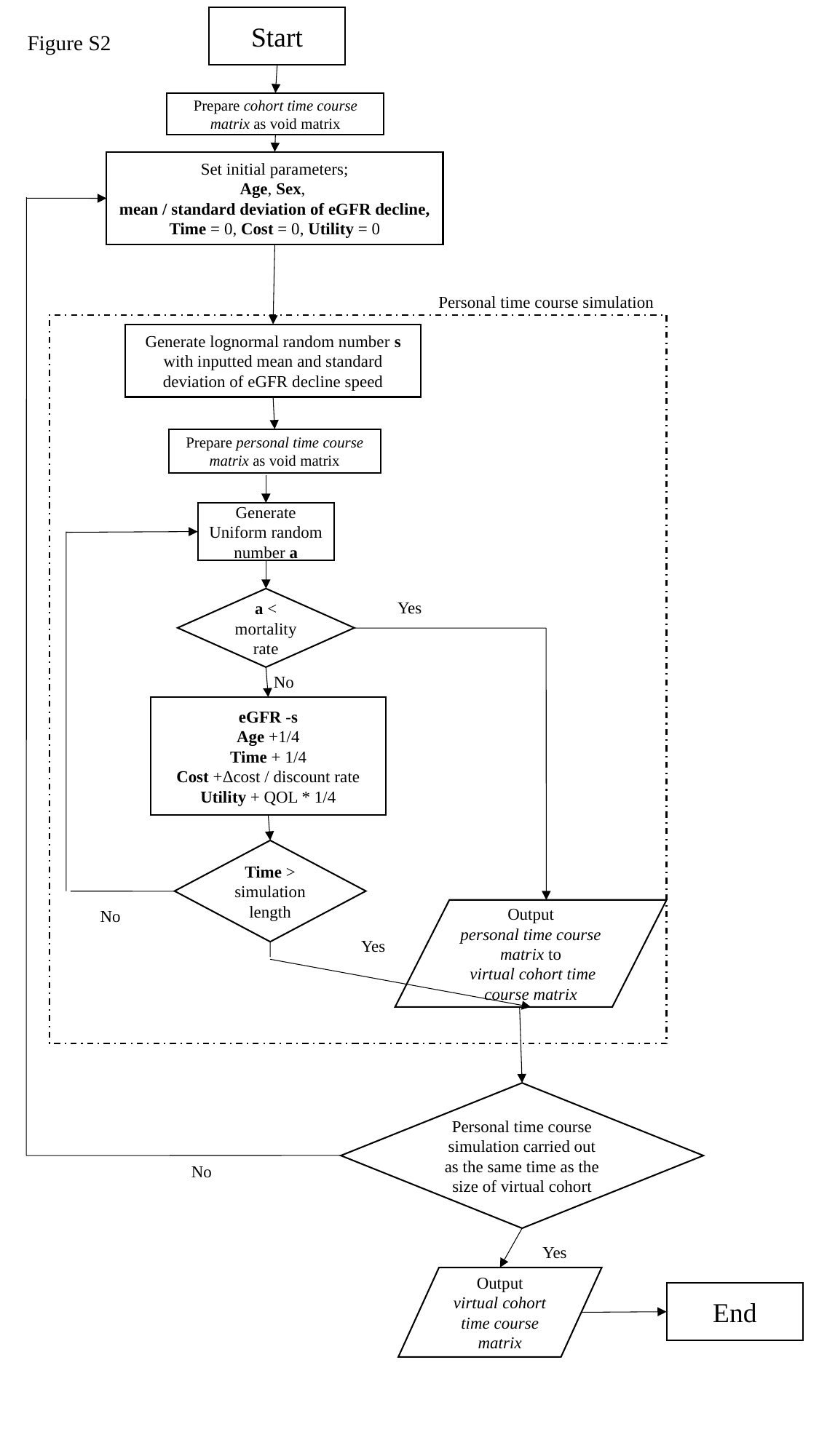

Start
Figure S2
Prepare cohort time course matrix as void matrix
Set initial parameters;
Age, Sex,
mean / standard deviation of eGFR decline, Time = 0, Cost = 0, Utility = 0
Personal time course simulation
Generate lognormal random number s
with inputted mean and standard deviation of eGFR decline speed
Prepare personal time course matrix as void matrix
Generate Uniform random number a
a < mortality rate
Yes
No
eGFR -s
Age +1/4
Time + 1/4
Cost +Δcost / discount rate
Utility + QOL * 1/4
Time > simulation length
No
Output
personal time course matrix to
 virtual cohort time course matrix
Yes
Personal time course simulation carried out as the same time as the size of virtual cohort
No
Yes
Output
virtual cohort time course matrix
End
