# Supplementary material for: The effect of model selection on cost-effectiveness research: a comparison of kidney function-based microsimulation and disease grade-based microsimulation in chronic kidney disease modeling
Source: BMC Med Inform Decis Mak. 2018 Nov 9;18:94. doi: 10.1186/s12911-018-0678-7 (PMC6230230; doi:10.1186/s12911-018-0678-7)

**Additional file 3**

**1. Determining virtual cohort size**

To make simulation results precise, it is essential to make the virtual cohort size larger, which means performing the simulation more times. However, simulation takes time in proportion to the virtual cohort size. Therefore, balancing simulation precision and calculation time is essential.

To determine the virtual cohort size, we began by examining the relationship between the virtual cohort size and calculated life-years (mean and standard deviation) using parameters from the VALIGA cohort described in the main text. The results converged toward one value as the cohort became larger, as shown in figure A-D attached below. Based on this result and our limited computer resources, we set the virtual cohort size as 30,000 in subsequent analyses, above which the life-years did not fluctuate more than 0.15 years in either model.

**2. Calculating mortality rates for validation**

As discussed in microsimulation implementation sections, our model used mortality rates as functions of age, sex and CKD grade. In validation simulation, we used data from VALIGA study, which included patients from 13 European countries. However, it is difficult to acquire life tables of included countries to calculate mortality rates, we instead used data from Ontario, Canada with the reason of similar ethnic composition. We established baseline mortality rates of　3.338 * 10^-5 * e^(0.091 * age) (men) and 1.615 * 10^-5 * e^(0.098 * age) (women) calculated using exponential approximation of data from life tables.

Mortality rates for virtual patients with CKD were set 1.2, 1.8, 3.2, and 5.9 times higher than the baseline for grades 3a, 3b, 4, and 5 before dialysis, respectively, adhering prior study [32].

The mortality rates of patients on dialysis in Europe were also difficult to obtain; therefore, we used data from the Japanese Society of Dialysis Therapy in 2013. Using this data, the mortality rate for patients on dialysis was set at　1.32 * 10^-3 * e^(0.060 * age)

**Figure A**:

**Figure B:**


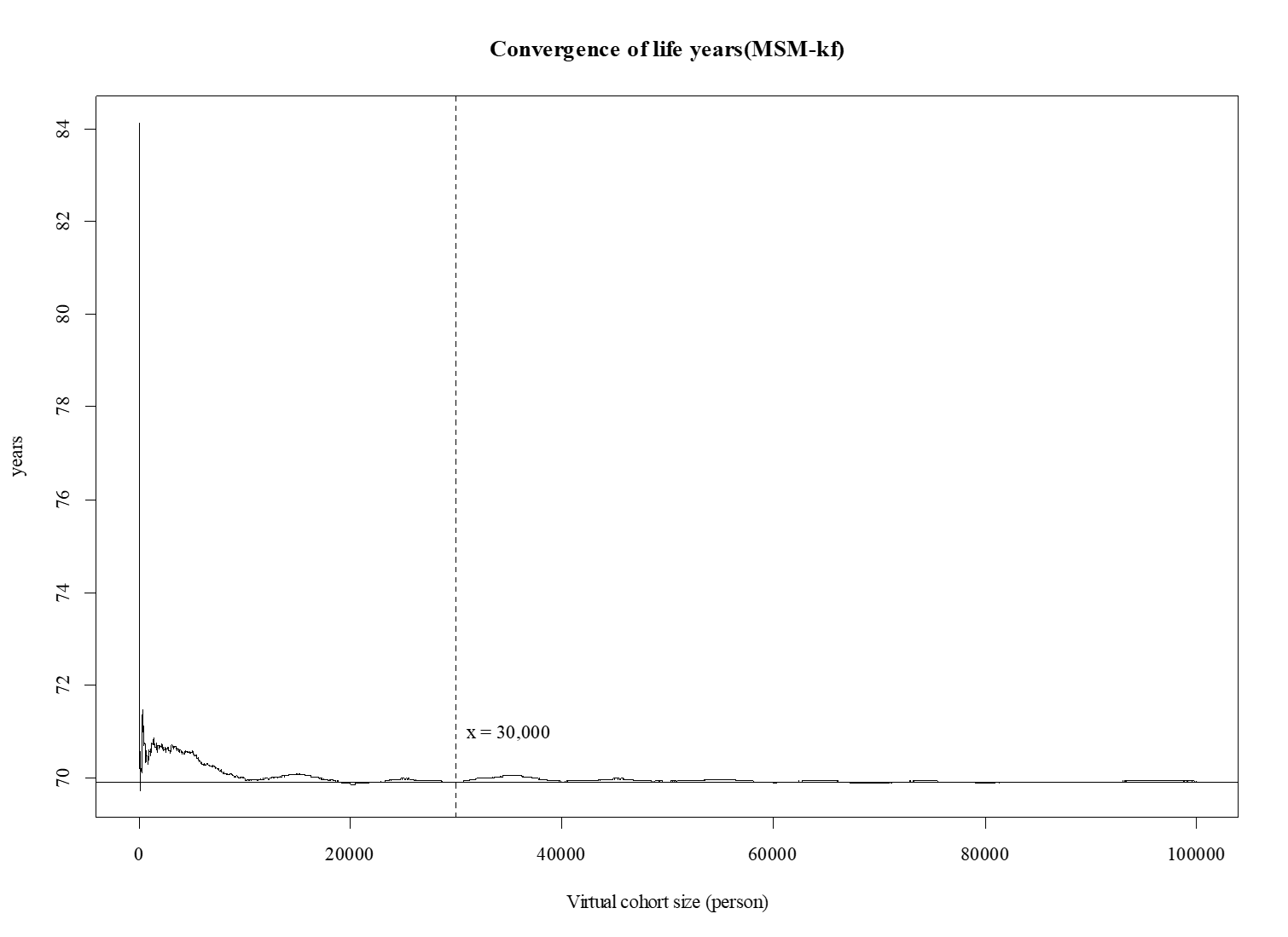


**Figure C:**


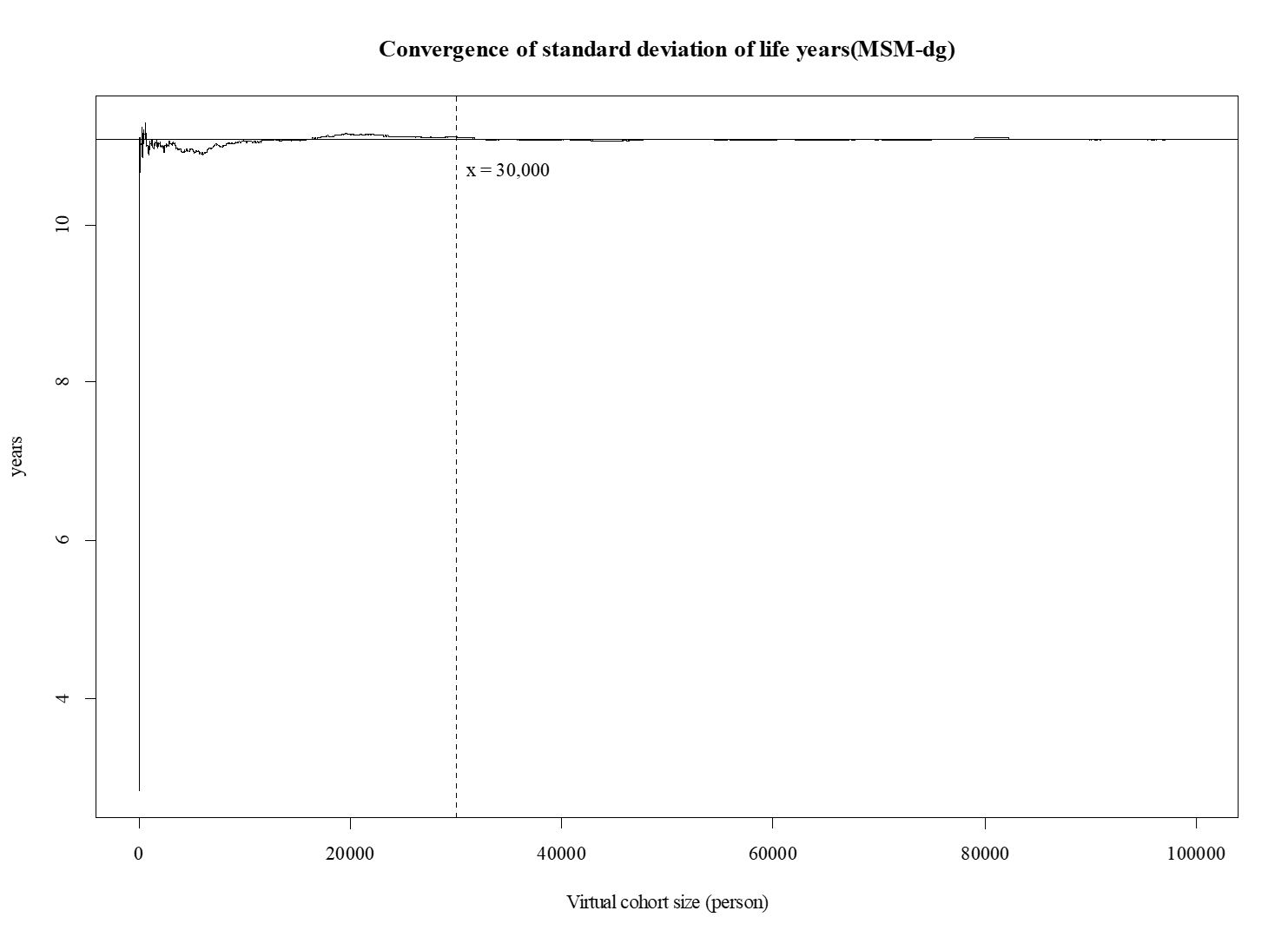


**Figure D:**


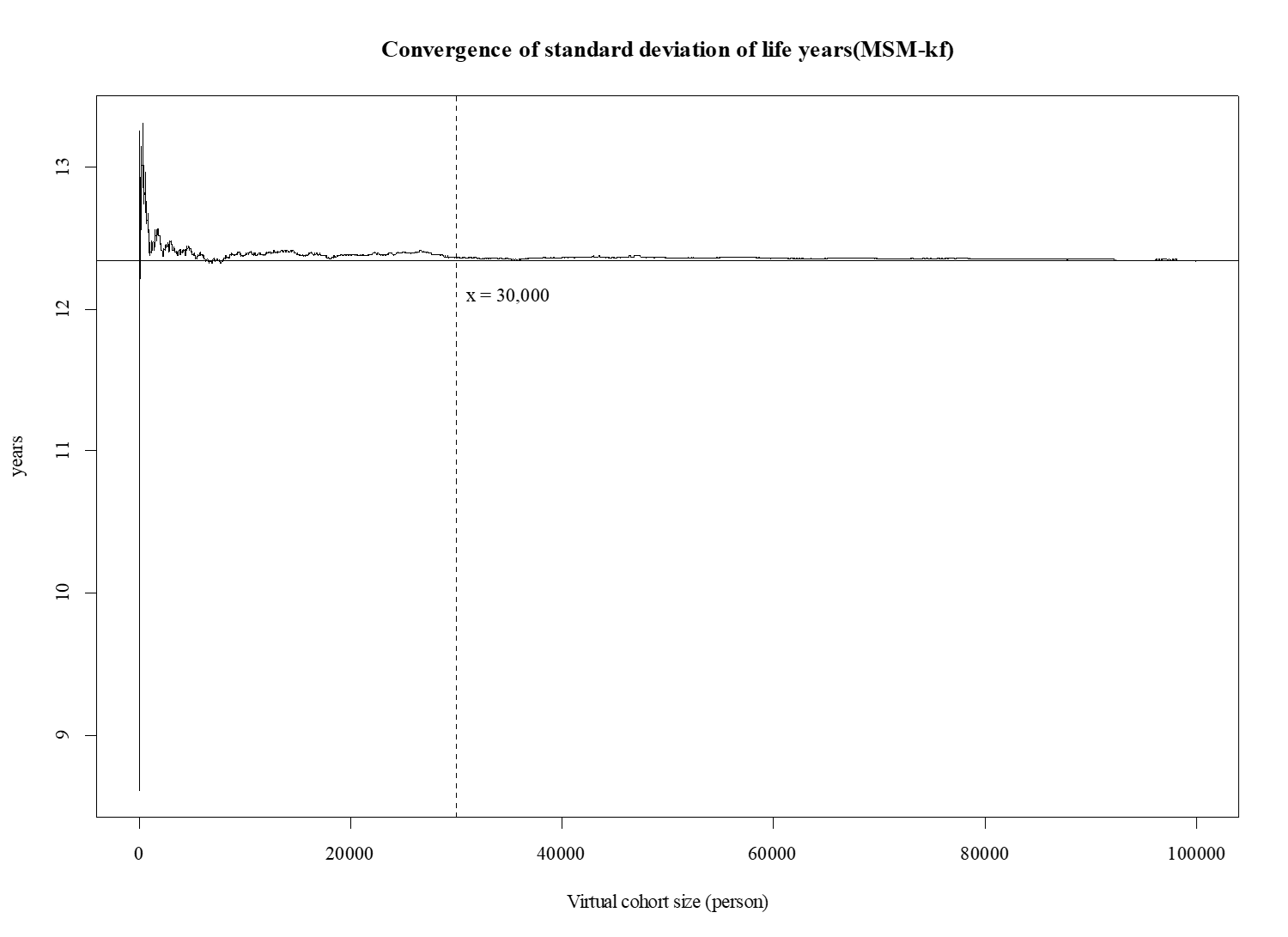

Supplement: Supplementary file 3 — Figure S2. Supplementary documents describing parameter determination in detail. (DOC 128 kb) [file 12911_2018_678_MOESM3_ESM.doc]
