# Supplementary figures and images for: The effect of model selection on cost-effectiveness research: a comparison of kidney function-based microsimulation and disease grade-based microsimulation in chronic kidney disease modeling
Source: BMC Med Inform Decis Mak. 2018 Nov 9;18:94. doi: 10.1186/s12911-018-0678-7 (PMC6230230; doi:10.1186/s12911-018-0678-7)

## Slide 1
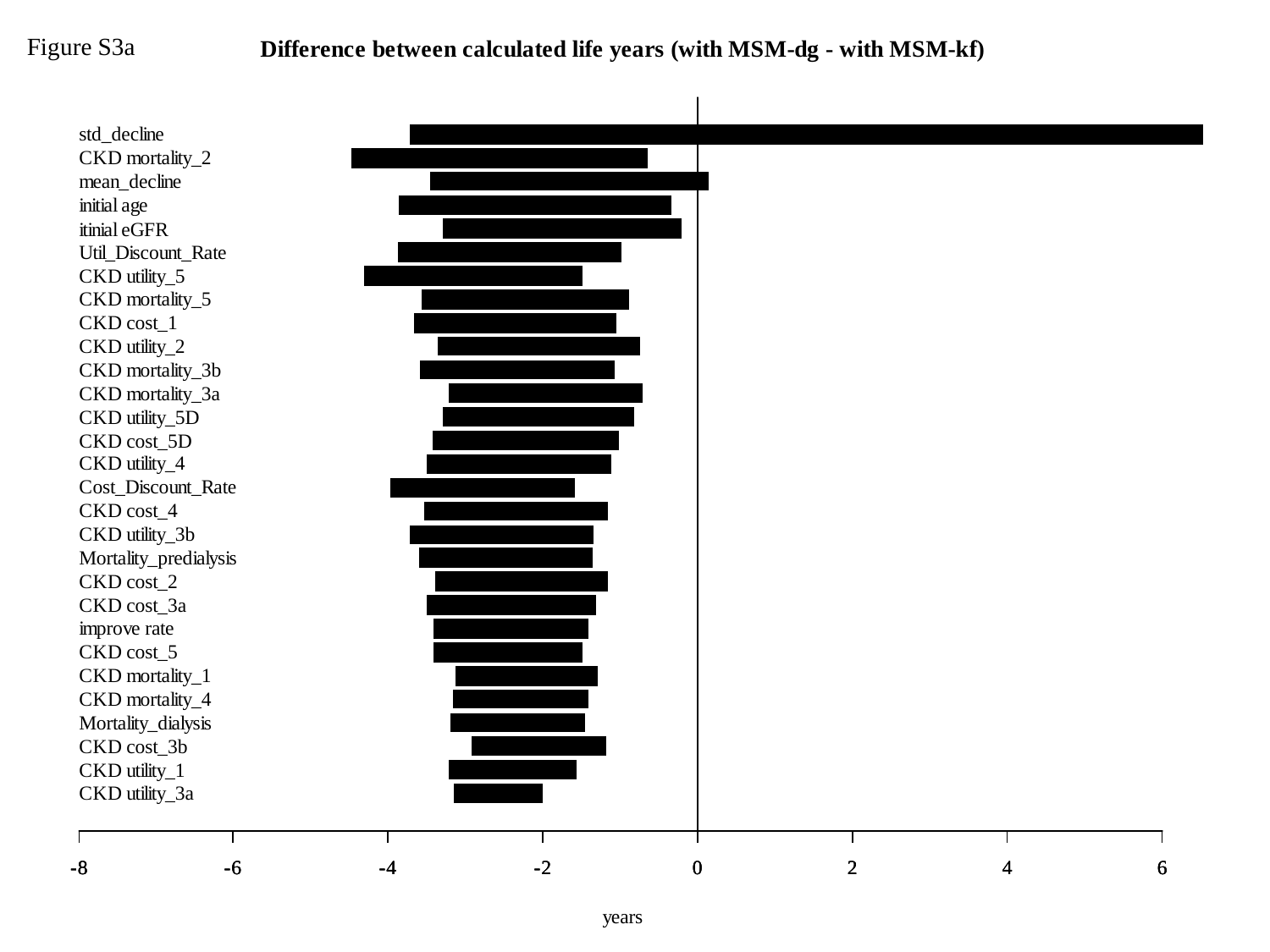

Figure S3a

## Slide 2
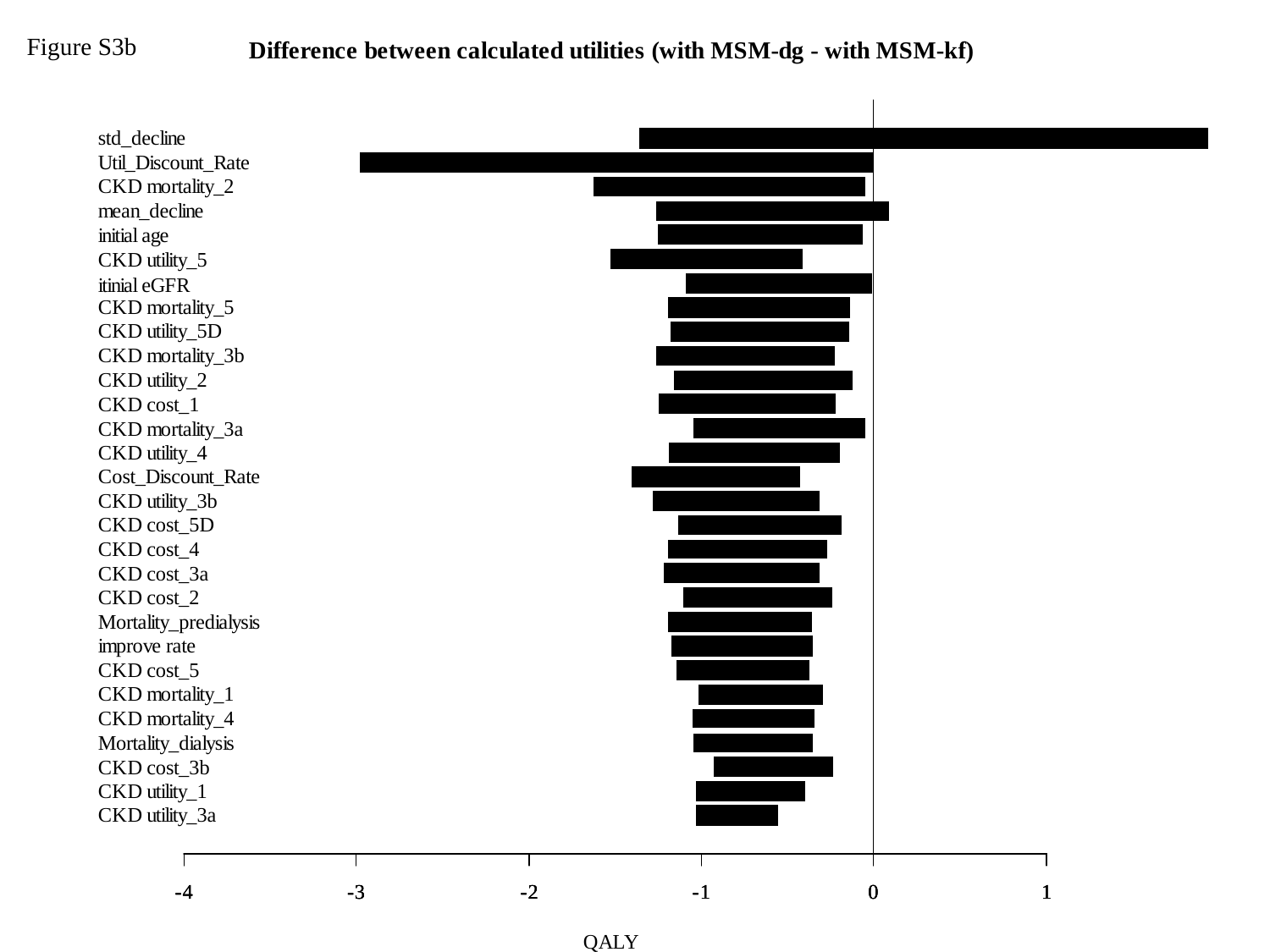

Figure S3b

## Slide 3
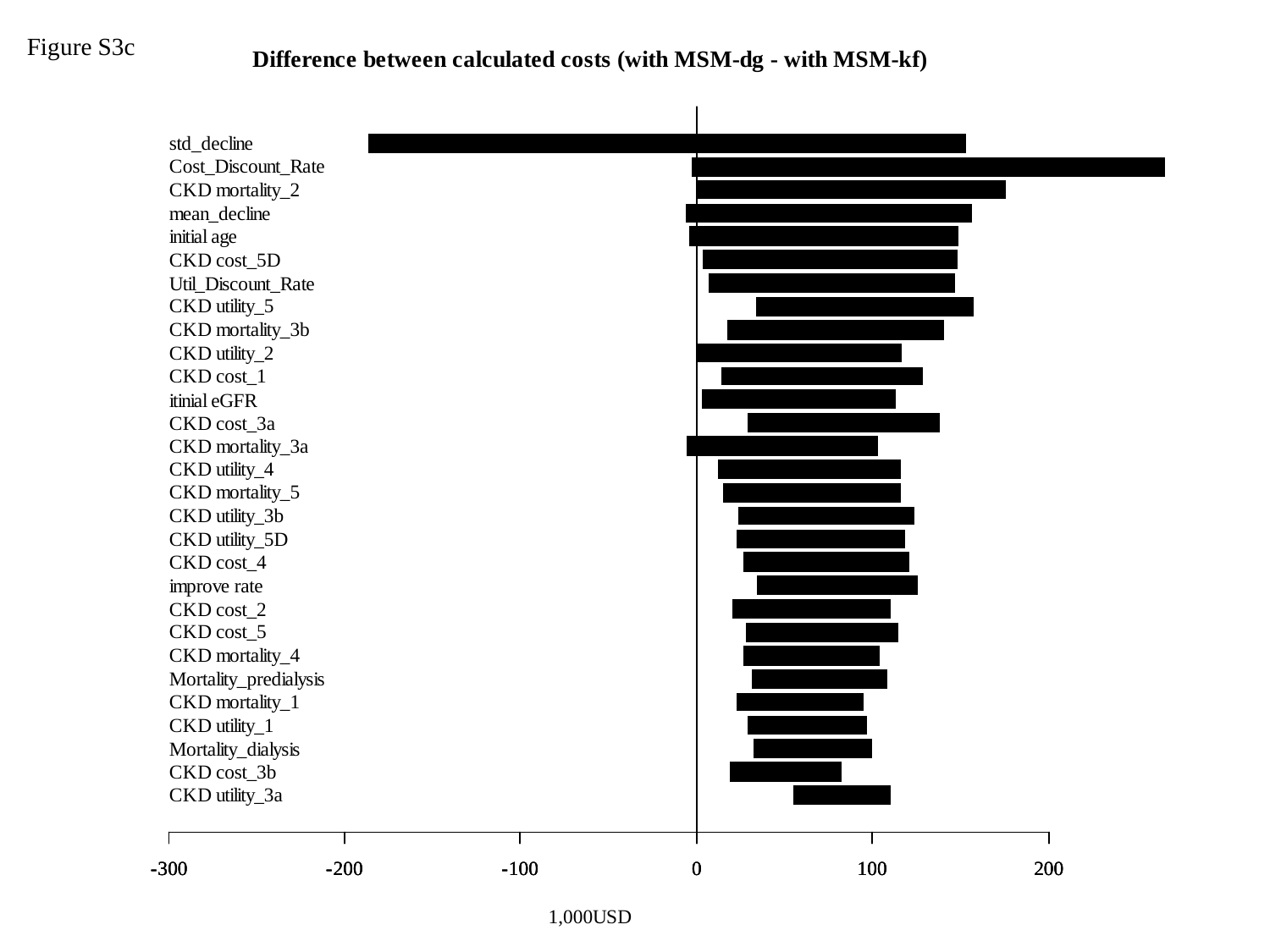

Figure S3c

## Slide 4
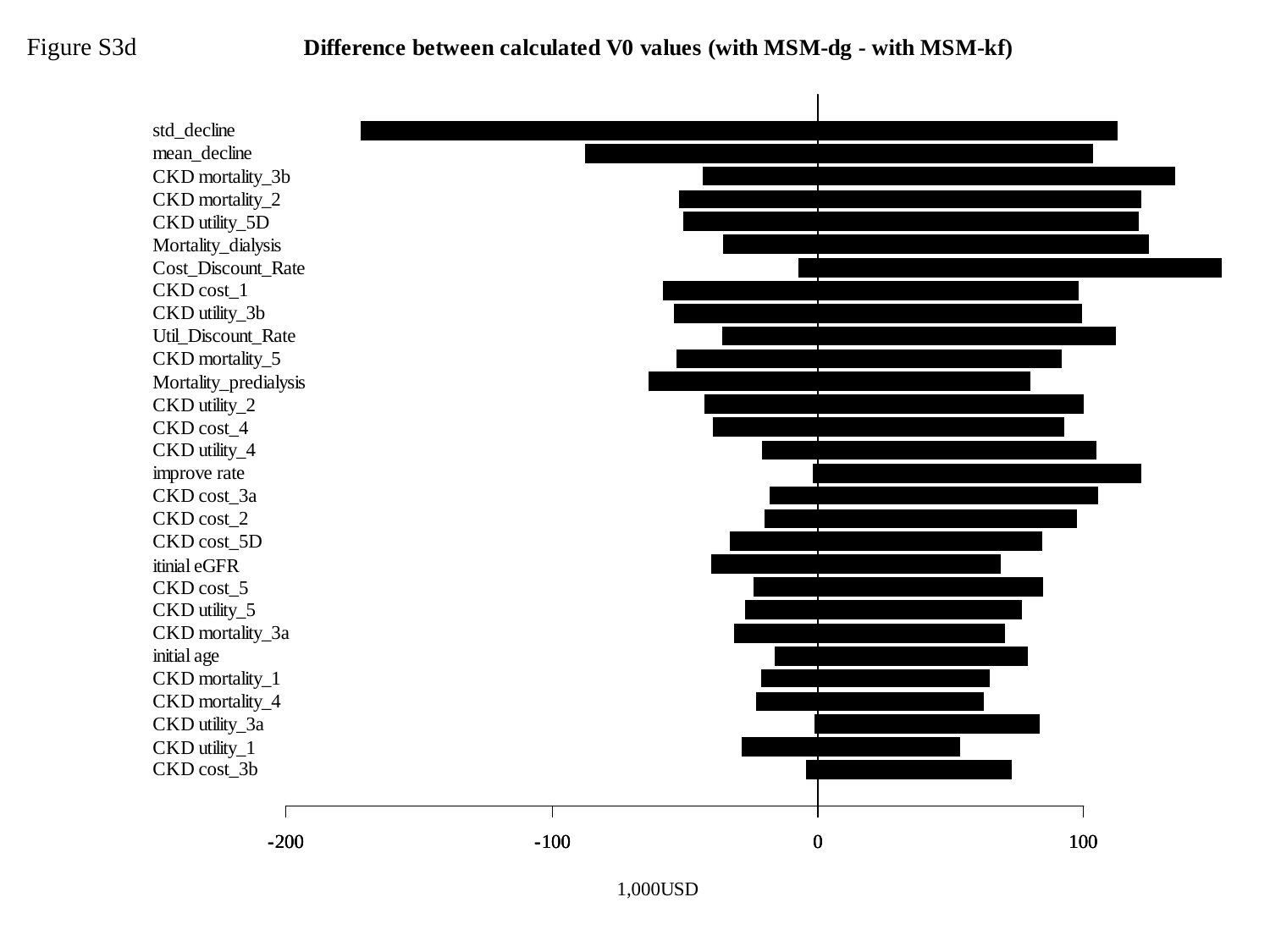

Figure S3d

## Slide 5
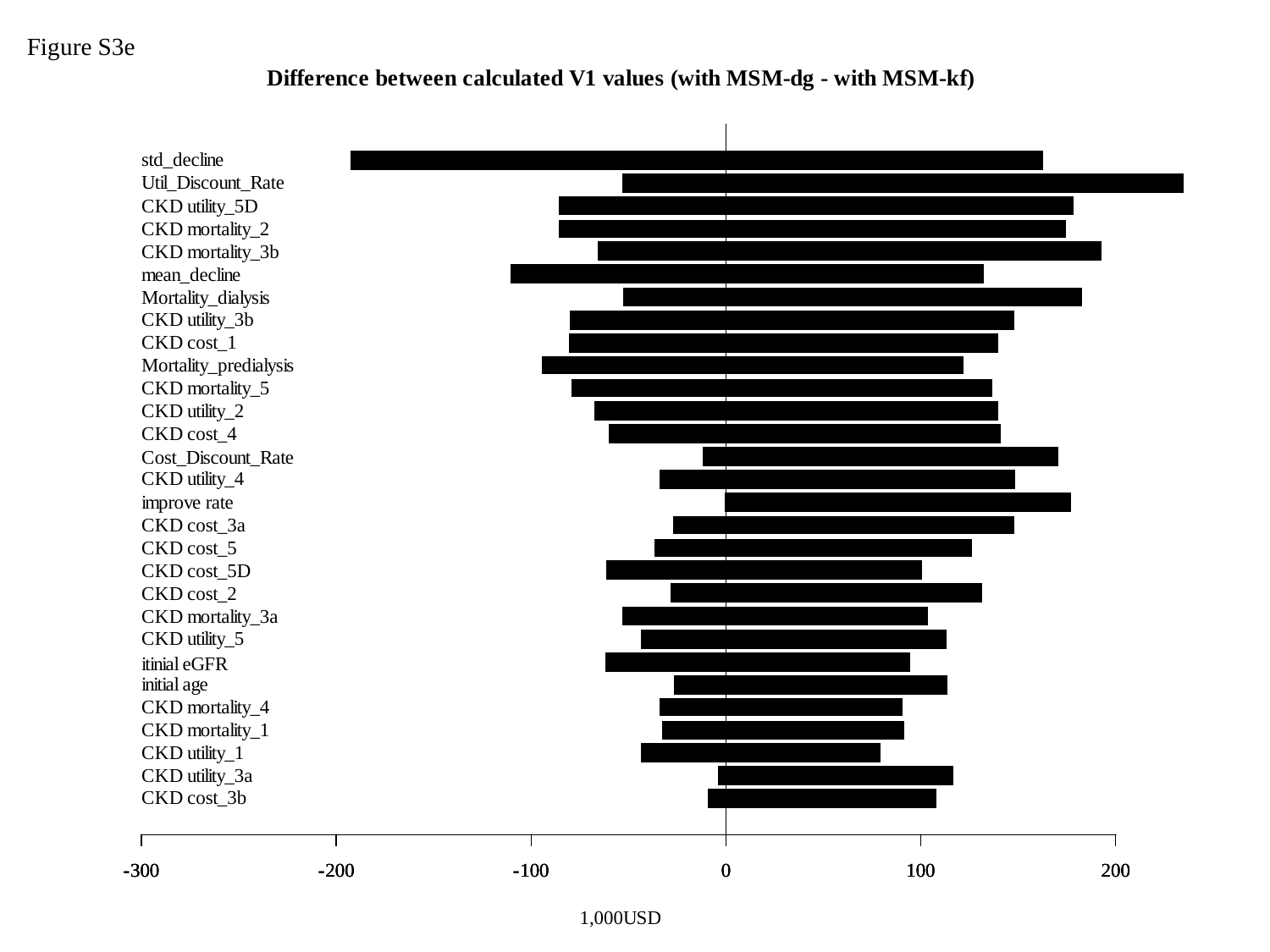

Figure S3e

Supplement: Supplementary file 6 — Figure S3. a-e Results of one-way sensitivity analyses. (PPT 132 kb) [file 12911_2018_678_MOESM6_ESM.ppt]

## Slide 1
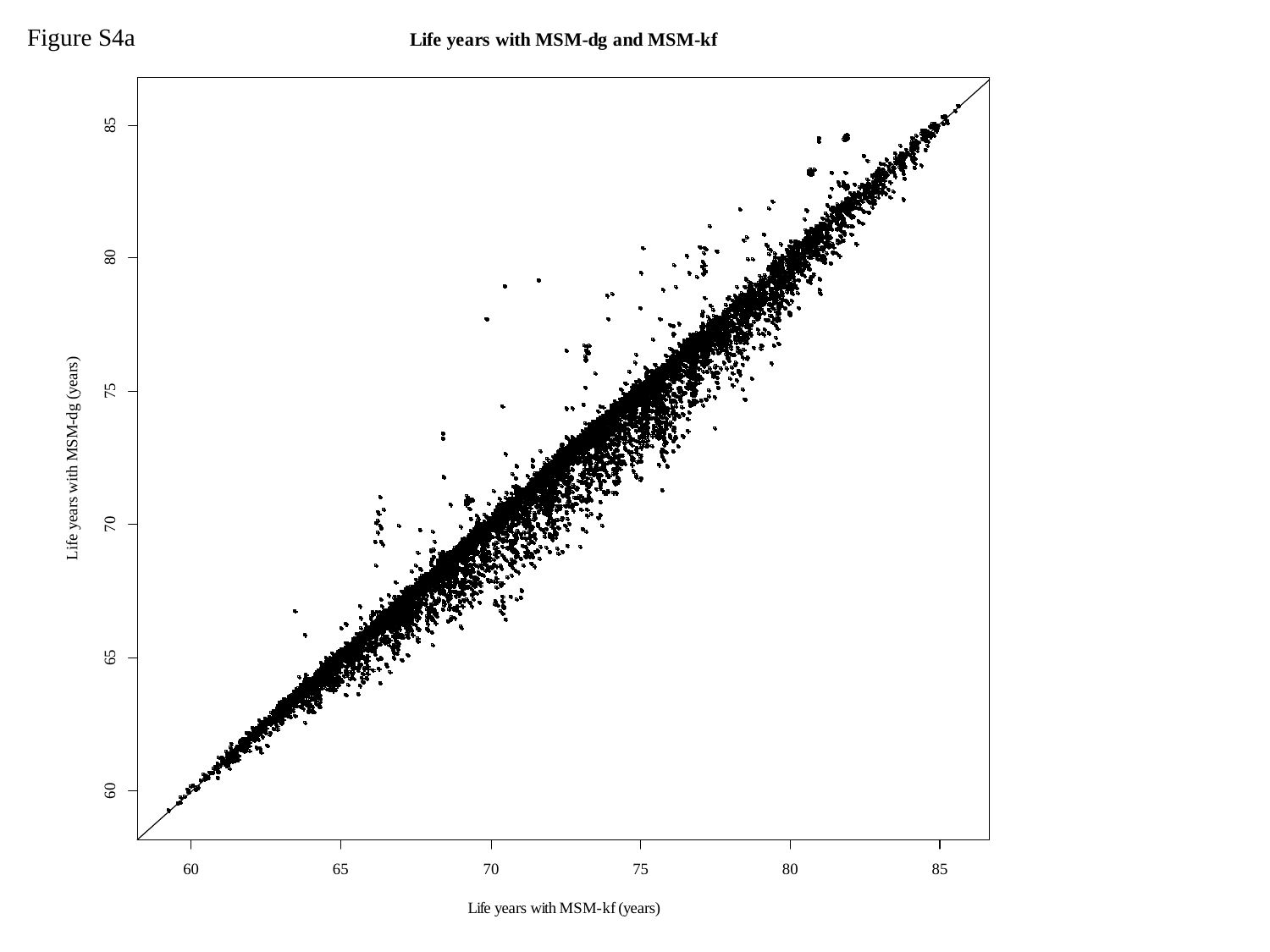

Figure S4a

## Slide 2
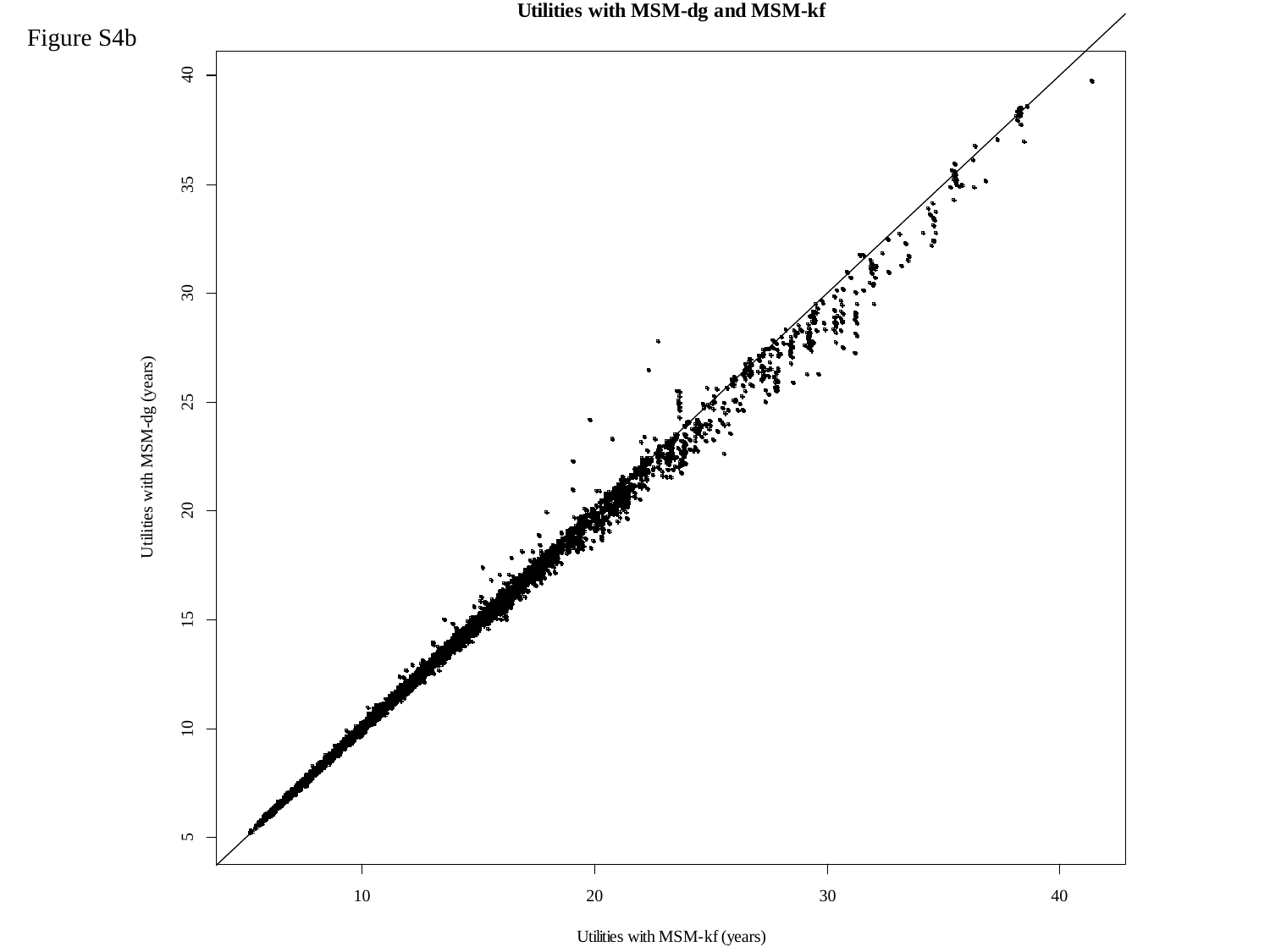

Figure S4b

## Slide 3
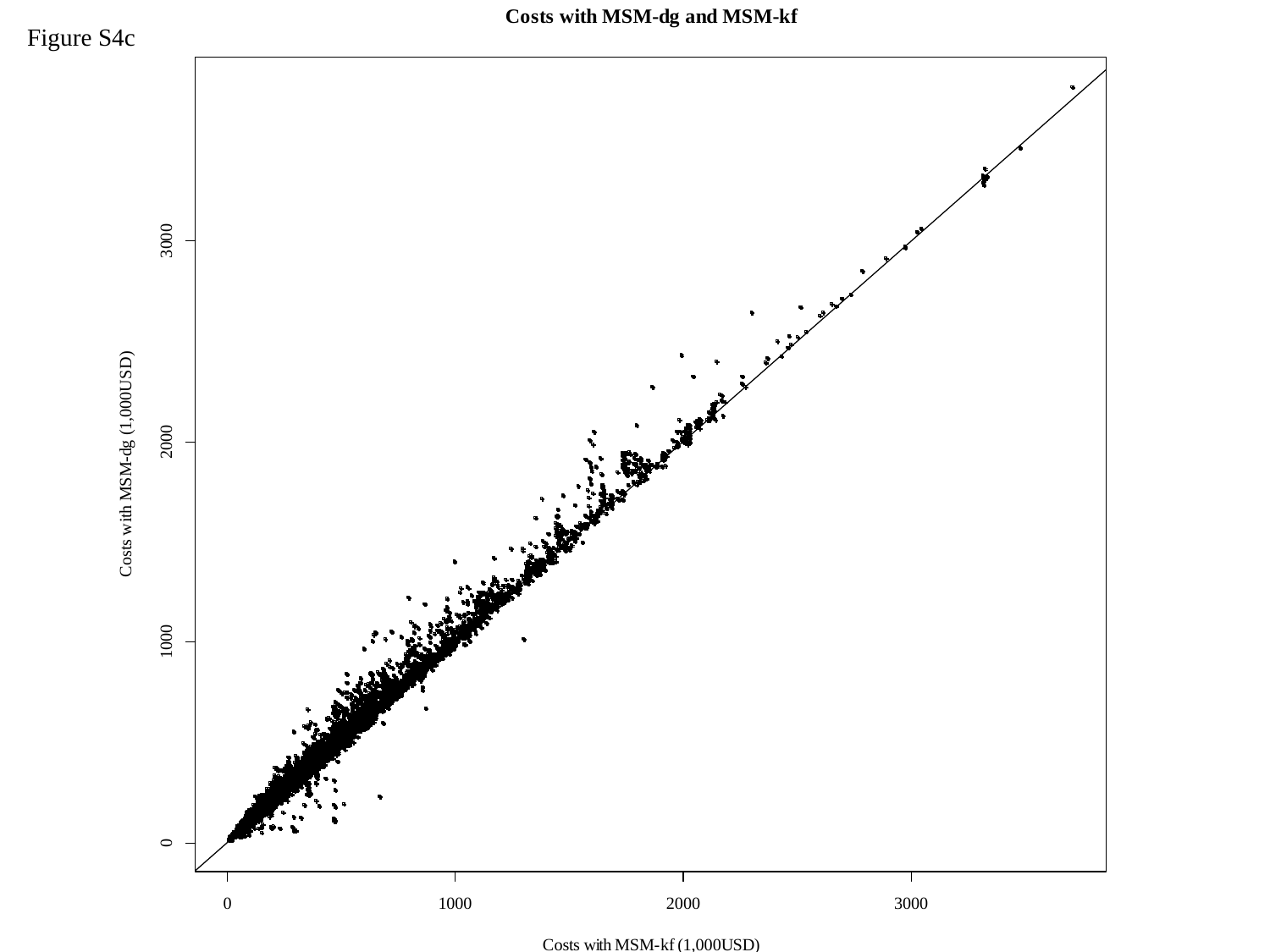

Figure S4c

## Slide 4
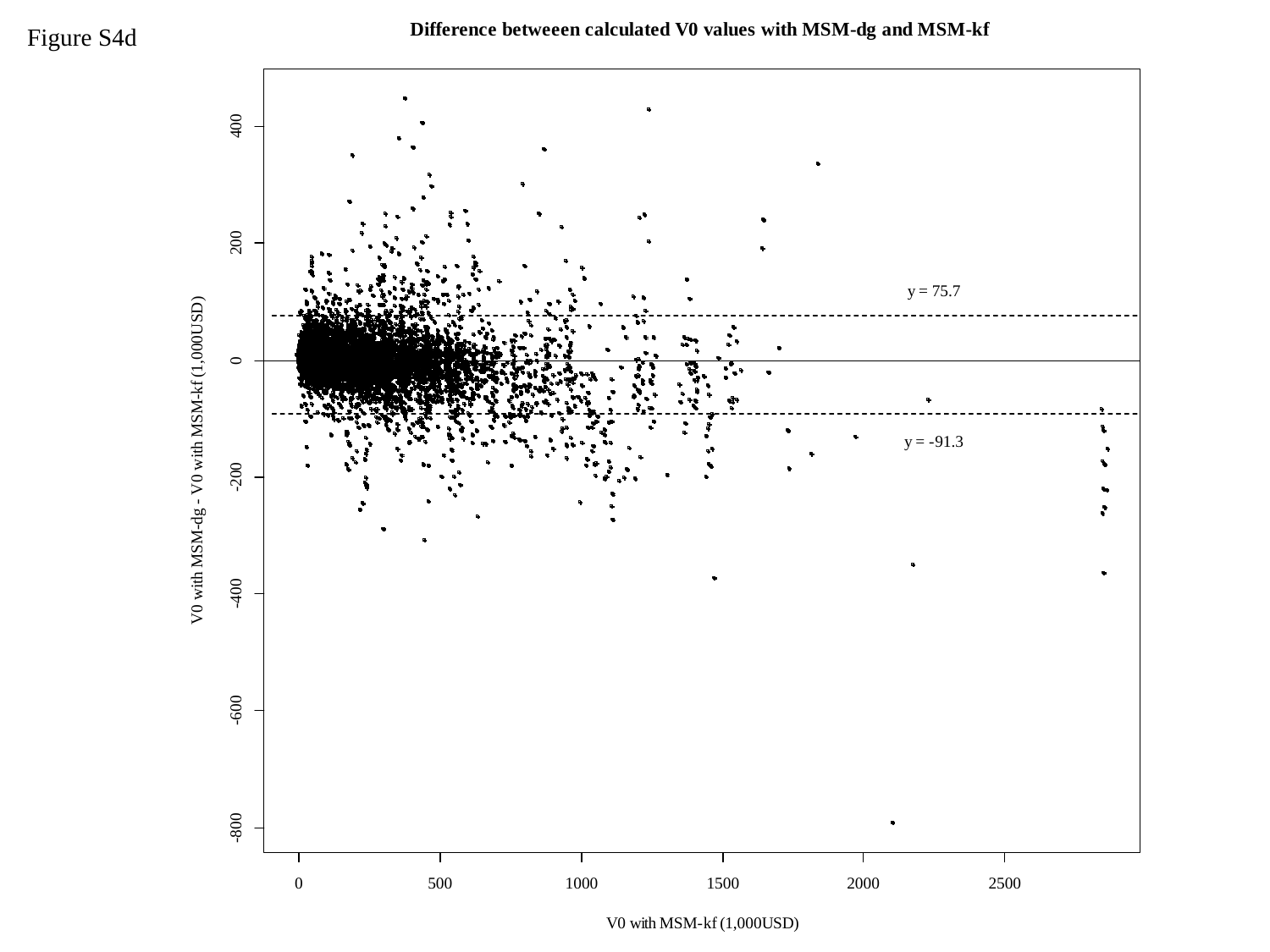

Figure S4d

## Slide 5
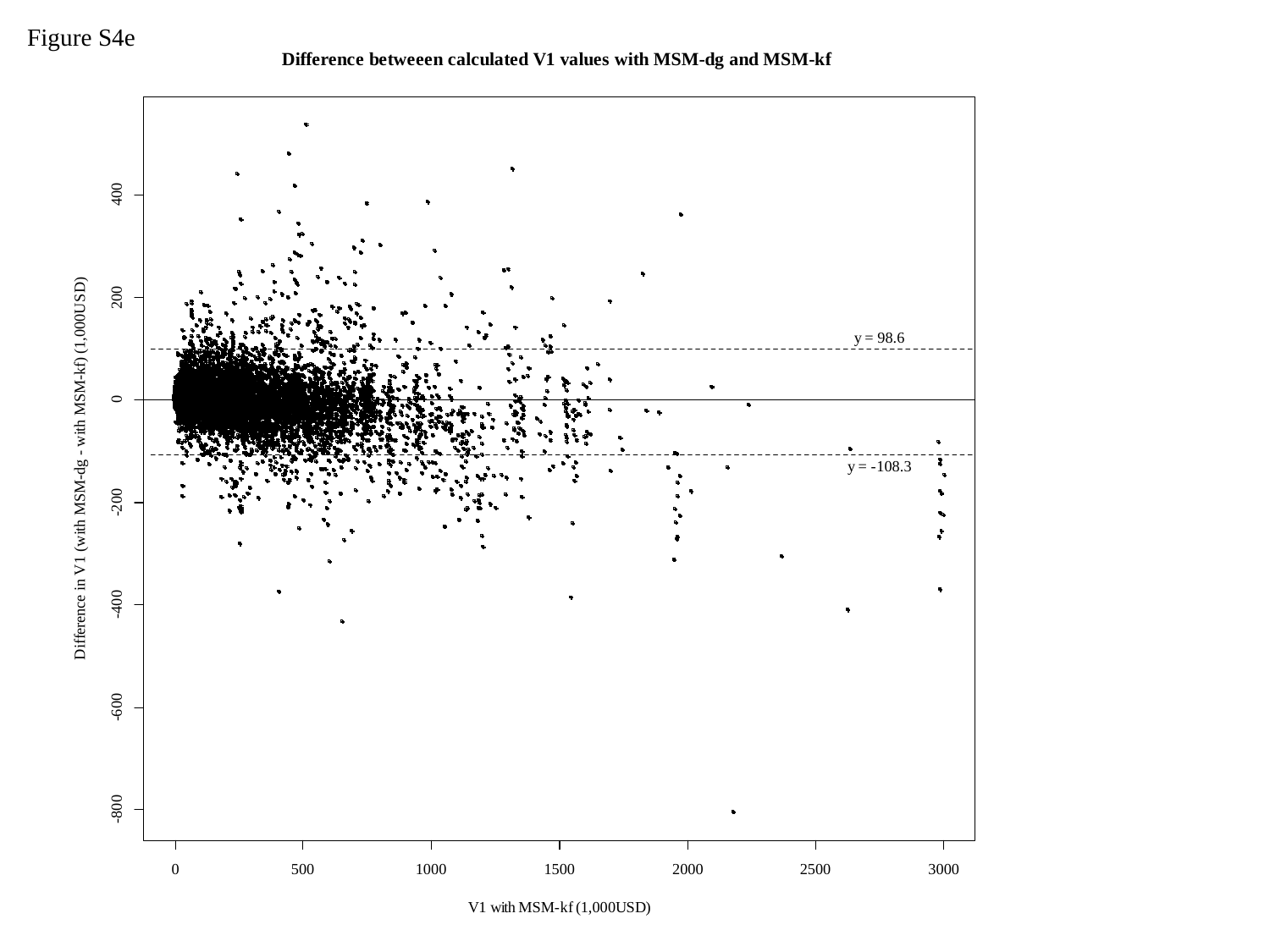

Figure S4e

Supplement: Supplementary file 7 — Figure S4. a-e Results of probabilistic sensitivity analyses. (PPT 3755 kb) [file 12911_2018_678_MOESM7_ESM.ppt]
